# The Threat of Zika Virus in Sub-Saharan Africa – The Need to Remain Vigilant

**DOI:** 10.3389/fpubh.2016.00110

**Published:** 2016-05-31

**Authors:** Vito Baraka, Eliningaya J. Kweka

**Affiliations:** ^1^Tanga Research Centre, National Institute for Medical Research, Tanga, Tanzania; ^2^Global Health Institute, Gouverneur Kinsbergen Centrum, University of Antwerp, Wilrijk, Belgium; ^3^Division of Livestock and Human Diseases Vector Control, Tropical Pesticides Research Institute, Arusha, Tanzania; ^4^Department of Medical Parasitology and Entomology, School of Medicine, Catholic University of Health and Allied Sciences, Mwanza, Tanzania

**Keywords:** arboviral infections, Zika virus, diagnosis surveillance and control, sub-Saharan Africa, commentary

## Background

News of the recent outbreak of Zika virus (ZIKV) disease in South America, North America, and Europe has generated great interest in the scientific community and general public like (1, 2). The disease is caused by the Zika virus (ZIKV), a mosquito-borne *Flavivirus* transmitted mainly by *Aedes aegypti* and *Aedes albopictus*, mosquitoes that also transmit other viral infections, including dengue virus (DENV), chikungunya virus (CHIKV), and yellow fever virus (YFV) (2). Zika virus was isolated for the first time in Rhesus monkey in 1947 in Zika forest in Uganda and since then, evidence of seroprevalence of ZIKV infection in human has been documented in several African countries (3–5). However, to date, the virus has not been considered a serious threat in the region. Symptoms of Zika virus disease are very similar to those of dengue and chikungunya and include fever, rash, joint pain, or conjunctivitis (6). Furthermore, most of the infections remain asymptomatic; thus, majority of the cases are either misdiagnosed or not detected at all. Worryingly, the recent pandemic in South America has associated ZIKV infection with microcephaly, a condition that results in small heads and underdeveloped brains in infants and neurological complication (Guillain–Barré syndrome); yet, no specific treatment or vaccine for the disease exists (1, 7). The incidences of ZIKV infections are escalating at alarming rates in South, North America, and in Europe and potentially threatening countries in sub-Saharan Africa if migration might play a role in both directions. Increasing urbanization, poor urban planning, changes in climatic factors, and the availability of favorable microecological condition suitable for *Aedes* mosquitoes breeding in sub-Saharan Africa are among factors that escalate mosquito abundance. In the face of such potential threat, there is a need for vigilance and establishment of preparedness measures before a Zika pandemic hits the continent. Such a pandemic would pose overwhelming cost burdens to the health systems and potentially compromise the achievement of the sustainable development goals (SDGs). In this letter, we wish to highlight measures that we believe would be effective in setting up countries’ preparedness response and surveillance systems to address the potential Zika virus disease threat in sub-Saharan context.

First, there is need for capacity strengthening with focus on the laboratory facilities and human resources to be able to implement epidemiological surveillance and disease control carry out accurate diagnosis and offer quality case management during outbreaks. The establishment of guidelines for all these aspects of disease management would need to be developed. There is need to support the setting up public health laboratories and strengthening of the existing ones to be able to conduct epidemiological surveillance and sophisticated molecular diagnosis that relies on polymerase chain reaction (PCR) or real-time PCR (RT-PCR) based assays. Currently, there is no ZIKV rapid diagnostic test available at the point of care. Therefore, it is important that health-care professionals are trained on case diagnosis and management approaches. This has to be in parallel with the improved capacity of laboratories to exclude other severe conditions, such as malaria and bacterial infection. Regional and cross-border networks, such as the East Africa Public Health Laboratory Networking (EAPHLNP), should be strengthened to fill the gaps and the model emulated in other African countries. In this era of increased mobility between countries, the need for regional coordination in sharing of virologic/serotype and vector surveillance data should be underscored. Additionally, there is a shortage of entomologists at regional and district levels to provide technical support in mosquito vector identification and dynamics, which is critical for vector surveillance and control. This shortage of qualified entomologists with technical field expertise is well documented (8). We therefore suggest that serious consideration be given to the training of entomologists so as to fill this gap.

Second, there is a need to raise community awareness and to educate the public on measures that they can put in place to avoid mosquito bites and reduce mosquito breeding habitats. *A. aegypti* are container breeders and integrated approaches that require close community engagement are necessary for their effective control. Community awareness and education will contribute toward the adjustment of risky behavior, such as the failure to cover water storage containers that are in use and improper disposal of old water containers and used car tires. The media should be granted the opportunity by governments to take the lead in supporting community awareness and education efforts on all matters related to epidemiology and control of Zika virus disease more than is currently happening (2).

Third, the role of global travel in the emergence and re-emergence of disease diseases cannot go unnoticed. There is already risk for transmission of Zika virus disease to sub-Saharan African countries in Cape Verde and other regions (3, 9). Expanding global travel and the shipping industry contribute significantly in the transportation of asymptomatic individuals (10). Strong incentives are needed for surveillance to prevent re-infestation of the Zika virus in sub-Saharan Africa. Attention is particularly needed in the main entry points such as the airports and seaports that are the main gateway from the infected areas. There is need for ministries of health and respective authorities to issue travel alerts and guidance to those visiting to ZIKV-risk countries. For example, pregnant women in any trimester should be advised to highly consider postponing travel and individuals who must visit such countries provided with guidelines and recommendations on the symptoms to look out for and immediately report to the health-care professional on the onset of such symptoms. Also, guidelines and recommendations on personal protection measures to avoid human–mosquito contact, such as the use of repellants and wearing long sleeve clothes, should be emphasized.

**Figure 1:**
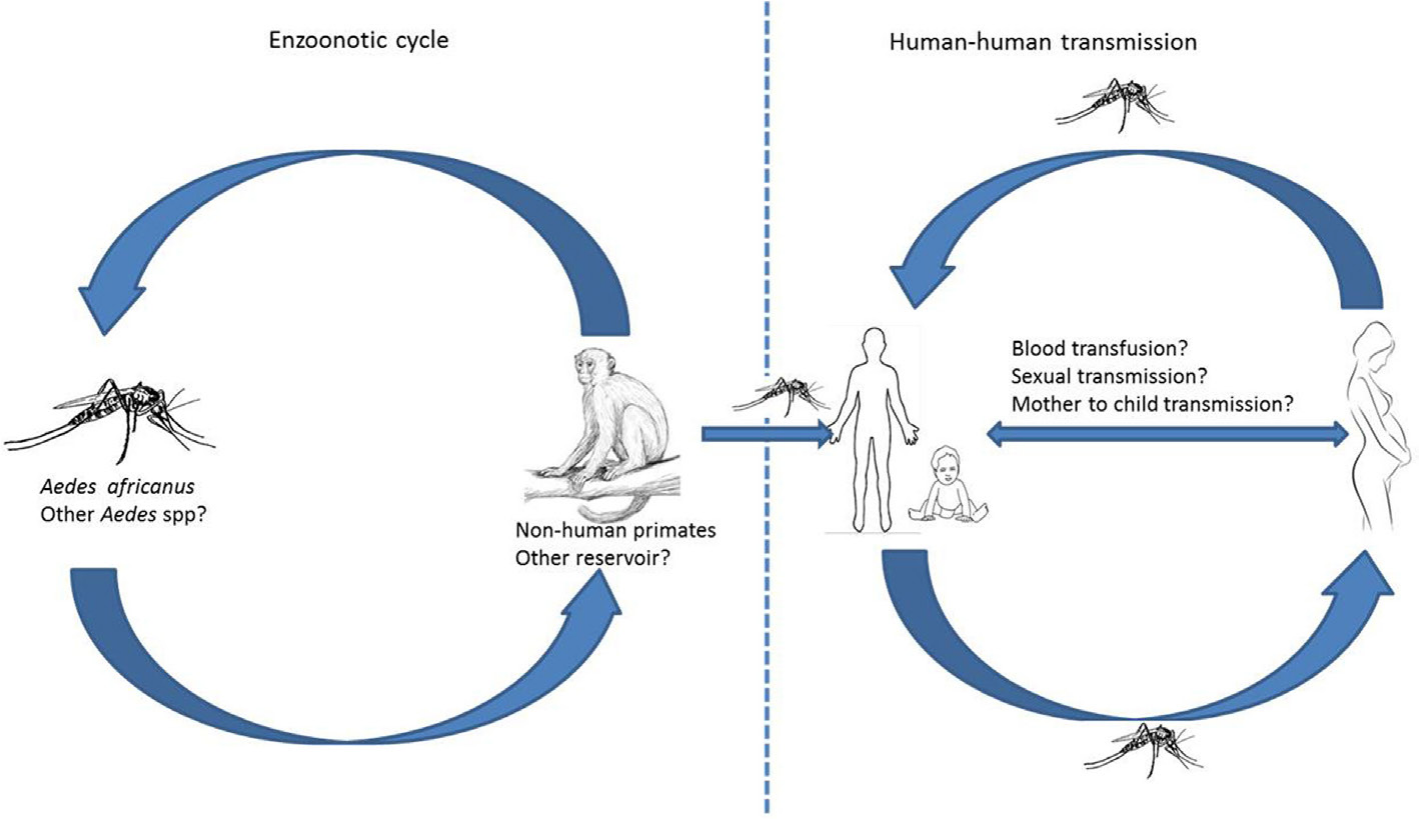
**Zika virus is transmitted mainly by the *Aedes aegypti* mosquitoes, which is widespread in urban and peri-urban areas**. The zoonotic is known to occur between human and non-human primates. The role of other reservoir and sexual transmission is still unconfirmed.

Fourth, several key research issues need to be addressed. It will be important to evaluate the role of potential non-human primates in maintaining transmission and/or serving as ZIKV reservoirs. Other proposed routes of transmission, including sexual and maternal (Figure 1), also need further investigation as this will have implications for the epidemiology of the disease. In addition, given that there are no preventative or therapeutic vaccines and point of care diagnostic tests for Zika virus disease, it is important that financial resources to accelerate the discovery and clinical testing of these tools be urgently set aside and made available. With regard to vector control, strategies relevant to the local context are urgently needed to support effective preventive and control measures. The increasing role of climatic factors in relation to *Aedes* mosquito dynamics also needs further exploration as the changes in global temperatures and weather patterns could impact the transmission and spread of the virus. Possible consequences of coinfections between dengue serotypes (DENV 1–4) and ZIKV virus or coinfection between ZIKV and other prevalent infections in the continent, such as malaria and HIV, also need to be understood. Furthermore, the implication of different ZIKV serotypes in vulnerable groups’ particularly pregnant women and children are yet to be understood. With Africa increasingly opening up to the rest of the world due to human migration associated with tourism and business, it is imperative for countries to remain vigilant regarding the threat of the expanding arboviral infections.

The re-emergence and spread of arboviral infections could lead to devastating consequences on the human population, the health-care system and economic progress in the continent. It is crucial that countries establish harmonized and robust vector control and surveillance systems, which will include the setting up of regional preparedness plans in response to mosquito-borne viruses and investing in capacity building as well as creating community awareness. Investing in research in the development and validation of tools and strategies for the control of the viruses and in understanding of their epidemiology will also be critical.

## Author Contributions

Both authors, VB and EK contributed equally to the drafting and approved the final manuscript for submission.

## Conflict of Interest Statement

The authors declare that the research was conducted in the absence of any commercial or financial relationships that could be construed as a potential conflict of interest.
